# Psychosocial factors as predictors of quality of life in chronic portuguese patients

**DOI:** 10.1186/1477-7525-12-3

**Published:** 2014-01-09

**Authors:** Estela Vilhena, José Pais-Ribeiro, Isabel Silva, Luísa Pedro, Rute F Meneses, Helena Cardoso, António Martins da Silva, Denisa Mendonça

**Affiliations:** 1 Sciences Department, School of Technology, Polytechnic Institute of Cávado and Ave (IPCA), Barcelos, Portugal; 2 Doctoral Program in Biomedical Sciences, Institute of Biomedical Sciences Abel Salazar (ICBAS), University of Porto (UP), Porto, Portugal; 3 Institute of Public Health (ISPUP), University of Porto, Porto, Portugal; 4 Faculty of Psychology and Educational Sciences (FPCE), University of Porto (UP), Porto, Portugal; 5 Department of Political Science and Behavior, Faculty of Humanities and Social Sciences, University of Fernando Pessoa, Porto, Portugal; 6 ESTeSL Polytechnic Institute of Lisbon (IPL), Lisboa, Portugal; 7 UMIB/ICBAS and Hospital Santo António/CHP, Porto, Portugal; 8 Population Studies Department, Institute of Biomedical Sciences Abel Salazar (ICBAS), University of Porto, Portugal, Porto, Portugal

**Keywords:** Chronic diseases, MANCOVA, Predictors, Psychosocial variables, Subjective well-being, Quality of life

## Abstract

**Background:**

Chronic illnesses are diseases of long duration and generally of slow progression. They cause significant quality of life impairment. The aim of this study was to analyse psychosocial predictors of quality of life and of subjective well-being in chronic Portuguese patients.

**Methods:**

Chronic disease patients (n = 774) were recruited from central Portuguese Hospitals. Participants completed self-reported questionnaires assessing socio-demographic, clinical, psychosocial and outcome variables: quality of life (HRQL) and subjective well-being (SWB). MANCOVA analyses were used to test psychosocial factors as determinants of HRQL and SWB.

**Results:**

After controlling for socio-demographic and clinical variables, results showed that dispositional optimism, positive affect, spirituality, social support and treatment adherence are significant predictors of HRQL and SWB. Similar predictors of quality of life, such as positive affect, treatment adherence and spirituality, were found for subgroups of disease classified by medical condition.

**Conclusions:**

The work identifies psychosocial factors associated with quality of life. The predictors for the entire group of different chronic diseases are similar to the ones found in different chronic disease subgroups: positive affect, social support, treatment adherence and spirituality. Patients with more positive affect, additional social support, an adequate treatment adherence and a feel-good spirituality, felt better with the disease conditions and consequently had a better quality of life. This study contributes to understanding and improving the processes associated with quality of life, which is relevant for health care providers and chronic diseases support.

## Background

Living with a chronic disease is a demanding experience that can affect multiple aspects of an individual’s life, such as social, family and occupational functioning. The Centers for Disease Control and Prevention [1] define chronic diseases as non-communicable illnesses that are prolonged in duration, do not resolve spontaneously, and are rarely cured completely. Chronic diseases include heart disease, cancer, stroke, diabetes, arthritis, obesity, and others. They affect everyday life and require adjustment on multiple life domains: adjustment is defined as a response to a change in the environment that allows an organism to become more suitably adapted to that change [2,3]. Typically, chronic patients are responsible for the management of the psychosocial factors that contribute to their health-related quality of life (HRQL).

Health-related quality of life (HRQL) is a construct reflecting the impact of health on overall well-being [4]. The World Health Organization (WHO) has further identified physical health, psychological state, level of independence, social relationships and relationship to salient features of the environment as core dimensions in determining life quality (WHOQOL Group 1993). It goes beyond direct measures of population health, life expectancy and causes of death, and focuses on the impact health status has on quality of life. A related concept of HRQL is well-being, which assesses the positive aspects of a person’s life, such as positive emotions and life satisfaction [5].

Considering the influence of several factors, such as social support [6], optimism [7], personality factors [8] on HRQL, numerous studies have analysed associations between these factors with HRQL and with subjective well-being (SWB). Subjective well-being refers to people’s emotional and cognitive evaluations of their lives, and includes what people usually call happiness, peace, fulfilment, and life satisfaction [9].

Dispositional optimism is defined as the general expectation or belief in positive outcomes in the future [10]. It has been associated with a variety of positive outcomes related to well-being [11], such as self-esteem, low depression, low negative emotions, and life satisfaction [12]. It also has been linked to indicators of quality of life, such as good health [12]. Therefore, given the impact that this variable exerts on HRQL, it appears important to know the ways through which optimism operates.

Emotional circumstances have been linked with mental and physical functioning. The emotional experience is composed by two factors: positive affect and negative affect. Positive affect refers to the individual’s positive emotional states such as joy, interest, confidence and alertness. Negative affect refers to negative emotional states such as fear, sadness, anger, guilt, contempt and disgust [13]. Social support has been defined as “an avocation interpersonal process that is centred on the reciprocal exchange of information and in a specific context; it consists of emotional and instrumental support and can improve mental health” [14], pg. 5. Parker et al. [4] reported that social support was beneficial in helping individuals in stressful situations. Spirituality is defined in many ways and can differ according to different religions. It reflects a unique psychological dimension around which individuals organize their lives, goals, values and intentions. It offers hope and opportunities for personal growth, and enhances social support, conferring important benefits for chronically ill people. A spirituality orientation may ease living with health challenges [15-17]. Treatment adherence is defined as the extent to which behaviour of a person is consistent with health care recommendations [18].

The importance of quality of life in chronic diseases has been increasingly recognized, given its implications for patients’ well-being, the use of health resources and a variety of elements that are required for a successful everyday life [2]. Several studies [10-13,15,19-21] tend to analyse quality of life in a specific chronic illness group and focus only on one or two exploratory (predictors) variables. The question of which variables affect the new life of chronic diseases patients is still a matter of debate. It will be necessary to further investigate the role of psychosocial variables in predicting quality of life in chronic patients to optimize understanding of their relationships and to design better intervention programs [22].

The main purpose of this study was to identify psychosocial predictors of HRQL and SWB in chronic Portuguese patients with the disease under control. The analyses were performed for the global group of several chronic diseases and in subgroups of diseases, which were divided according to medical condition.

## Methods

### Patients and design

This cross sectional study included 774 chronic patients, having in common the fact that they have returned to everyday life after diagnosis and treatment prescription. These patients were approached directly by their physicians during the consultation in the outpatient departments of the central Portuguese Hospitals. Inclusion criteria were: 1) diagnosis of epilepsy, diabetes, multiple sclerosis, obesity, myasthenia gravis or cancer disease diagnosed at least 3 years prior the study; 2) age ≥17 years at the time of the interview; 3) educational level higher than 6 years; 4) had return to usual daily life with disease under control; 5) no cognitive disturbances. Prior to data collection, ethical approval for this study was obtained from the institutions’ ethical committees. After a description of the study aims and of participants’ rights, all patients who met the inclusion criteria agreed to participate.

## Measures

Patients completed self-reporting questionnaires to assess socio-demographic, clinical and psychosocial variables, quality of life - HRQL (three components: general well-being – GWB, physical health – PH and mental health – MH) and subjective well-being (SWB). Psychologists collected the data, after medical appointments. Severity of disease was assessed with an anchoring vignete scale [23] following the recommendations of Sen (2002) [24] and the Eurostat statistics report practices. The scale is similar to the pain severity scale [25].

### Socio-demographic and clinical variables

Socio-demographic and clinical information was obtained regarding sex, education, age, time since diagnosis and severity of disease perception (“generally, how do you classify your illness?” with an increasing scale from 1-nothing serious, to 11-very serious).

#### Psychosocial variables

##### Dispositional optimism

Dispositional optimism was evaluated with the Life Orientation Test-Revised (LOT-R) [26]. The LOT-R was developed to assess individual differences in generalized optimism (e.g. “In uncertain times, I usually expect the best”) versus pessimism (e.g. “If something can go wrong for me, it will”). Validation of the Portuguese scale [27] showed similar characteristics to the original version. It consists of ten statements, in which three items reflect expectations for positive outcomes, three items reflect expectations for negative outcomes, and four are filter items. The optimism score was calculated by adding the three optimism questions and the pessimism score was calculated by adding the three pessimism questions The overall LOT-R score (range: 6–30) was calculated by reverse scoring the three pessimism scores, and summing responses to all six questions. Higher scores mean a higher degree of dispositional optimism. The Portuguese version shows a Cronbach alpha of 0.71.

### Positive and negative affect

Affect was assessed using the Positive and Negative Affect Schedule (PANAS), constructed by Watson, Clark and Tellegen [28] which addresses both positive affect (PA) and negative affect (NA). The PANAS schedule was validated to the Portuguese population by Galinha and Pais Ribeiro [29]. The results revealed similar characteristics to the original version. Items were averaged to obtain scale scores (range: 1–5), and high scores of PA indicate more positive affect, or the extent to which the individual feels enthusiastic, active and alert. A higher score of NA indicates more negative affect, which reflects the individual’s aversive mood states and general distress. The Portuguese version found an internal consistency 0.86 for the positive affect and 0.89 for the negative affect scales.

### Spirituality

Pinto and Pais Ribeiro [30] developed a scale to evaluate the spirituality of the Portuguese population, which considers both religious/spiritual perceptions and the hope of the patient. The five items were rated on a Likert-type scale with the response options from "do not agree" to "strongly agree". The determination scores were obtained through elementary arithmetic procedures, without inversion or transformation of values (range: 1–4), with a resulting midpoint of 2.5 for each item. Therefore, when the scores assume a value above the midpoint, it can be affirmed that the dimension of spirituality is identified as relevant. For the global scale, the authors found an internal consistency of 0.74.

### Social support

For the Portuguese population social support was assessed with the Social Support Survey (MOS) [31-33]. This is a multidimensional self-questionnaire that evaluates various dimensions of social support. The MOS consists of four separate social support subscales: emotional/informational, tangible, affectionate, and positive social interaction. An overall functional social support index is also used (range: 0–100). All subscales have shown strong reliability over time with a Cronbach alpha higher than 0.91.

### Treatment adherence

To assess treatment adherence a Portuguese version of the questionnaire (Medida de Adesão aos Tratamentos), based on previous studies [34], was developed with seven items by Delgado and Lima [35]. The treatment adherence score is the mean of the items in which higher values mean better treatment adherence. The measure showed good internal consistency.

#### Outcome variables

##### Quality of life

Health status perception was measured with the Medical Outcomes Study MOS 36-item Short-Form Health Survey (SF-36) [36], a 36-item questionnaire divided into eight dimensions that represent two major components: the physical and the mental components of health. In this study, we used the general dimension Well-Being that results from the IQOLA project [37], in which a second-order factor was found, with three components of SF-36 (general well-being - GWB, physical health - PH and mental health - MH). Each scale is converted directly into a 0–100 scale on the assumption that each question carries equal weight, in which 100 represents the highest level. The Portuguese version of the MOS SF-36 [38,39] shows good levels of internal consistency (Cronbach α of 0.70).

### Subjective well-being

Subjective well-being was evaluated using the Portuguese version of the Personal Well-being scale, which includes seven areas (satisfaction with level of life, health, personal achievement, personal relationships, sense of safety, community connection and future security). The score is the average of the items, varying from 0 to 100, in which higher values represent better subjective well-being. The Portuguese version shows a Cronbach α of 0.81 [40].

## Statistical analysis

Descriptive statistics were calculated to assess sample characteristics. Patients were classified by medical conditions into three groups: metabolic diseases (obesity and diabetes, 47.2%), neurologic (epilepsy, multiple sclerosis and myasthenia gravis, 25.7%) and cancer (27.1%).

Pearson correlation was used to examine the associations between psychosocial and outcome (HRQL and SWB) variables.

Multivariate Analysis of Covariance (MANCOVA) was used to identify independent predictors of quality of life and of subjective well-being, controlling for socio-demographic and clinical variables. This analysis, which takes into account the possible association between outcome variables, included as potential predictors dispositional optimism, positive and negative affect, social support, spirituality and treatment adherence.

MANCOVA assumptions were evaluated. To achieve normality, physical and mental health components values were transformed into $\sqrt{\left(k+1\right)-\mathrm{value}}$, where k was the maximum value that the variable takes [41] in this sample. Significant level was set at 0.05. All analyses were carried out using the Statistical Package for Social Sciences (SPSS, Version 20.0).

## Results

### Participant characteristics

The sample included 774 chronic patients: 27.1% with cancer, 17.2% with diabetes, 12% with epilepsy, 2.2% with myasthenia gravis, 11.5% with multiple sclerosis and 30% with obesity. Socio-demographic and clinical characteristics of the group and subgroups of chronic disease patients are presented in Table 1.

**Table 1:** Socio-demographic and clinical characteristics by group and subgroups of chronic diseases

		**Total group of chronic diseases**	**Subgroups of chronic diseases**		
		**(n=774)**	**Metabolic (n=365)**	**Neurologic (n=199)**	**Cancer (n=210)**
**n (%)**					
Sex					
	Male	228 (29.50)	91 (24.90)	68 (34.20)	69 (32.90)
	Female	546 (70.5)	274 (75.10)	131 (65.80)	141 (67.10)
Education level					
	≥9 Years	465 (60.10)	187 (51.20)	161 (80.90)	117 (55.70)
	<9 Year	309 (39.90)	178 (48.80)	38 (19.10)	93 (44.30)
**Mean (SD)**					
Age (years)		42.98 (11.55)	42.93 (0.64)	36.52 (0.61)	48.80 (0.71)
Time since diagnosis (years)		12.82 (9.73)	14.19 (0.56)	14.44 (0.74)	9.01 (0.49)
Severity of disease perception		6.56 (2.81)	7.29 (0.15)	5.50 (0.18)	6.34 (0.21)

### Associations between psychosocial variables and HRQL and SWB

Table 2 displays the descriptive statistics and correlations between psychosocial variables and outcome variables (HRQL components and SWB). Negative affect showed a statistically significant inverse association with HRQL components and SWB. Dispositional optimism and positive affect had a significant positive association with HRQL components and SWB. Spirituality only had a significant positive correlation with SWB. The other psychosocial dimensions showed a statistically significant association with HRQL components and with SWB. Statistically significant correlations were also found between outcome variables.

**Table 2:** Descriptive statistics; correlations between psychosocial variables and HRQL components and SWB

	**Mean**	**SD**	**DOp**	**PA**	**NA**	**Sp**	**SS**	**TA**	**GWB**	**MH**	**PH**	**SWB**
**Psychosocial Variables**												
Disp. Optimism (DOp)	20.68	4.12	-	0.44*	−0.43*	0.32*	0.32*	0.18*	0.40*	0.38*	0.21*	0.42*
Positive Affect (PA)	2.94	0.78		-	−0.18*	0.24*	0.32*	0.11*	0.41*	0.33*	0.24*	0.36*
Negative Affect (NA)	1.96	0.75		-	-	−0.13*	−0.25*	−0.24*	−0.49*	−0.58*	−0.37*	−0.42*
Spirituality (Sp)	2.71	0.75				-	0.26*	0.05	0.20*	0.15*	0.17	0.41*
Social Support (SS)	68.55	21.37					-	0.14*	0.30*	0.32*	0.23*	0.34*
Treatment Adherence (TA)	5.41	0.54						-	0.21*	0.22*	0.20*	0.19*
**Quality of Life Variables**												
General Well-being (GWB)	49.84	18.90							-	0.66*	0.71*	0.56*
Mental Health (MH)	66.65	25.14								-	0.65*	0.50*
Physical Health (PH)	63.99	26.47									-	0.43*
Subjective Well-being (SWB)	63.60	17.07										-

*p<0.05.

### MANCOVA analysis

Considering the associations between outcome variables, and controlling for socio-demographic and clinical variables, further analysis was performed using MANCOVA.

A statistically significant association was found between each of the psychosocial variables with the HRQL components and SWB (Table 3).

**Table 3:** Psychosocial factors associated with HRQL and SWB: results of multivariate tests of MANCOVA analysis*

	**Value Roy’s largest root**	**F**_**(4, 667)**_	**p**	**Observed power**^**a**^
Dispositional Optimism	0.02	3.57	**0.007**	0.87
Positive Affect	0.08	13.01	**<0.001**	1.00
Negative Affect	0.32	53.45	**<0.001**	1.00
Spirituality	0.13	21.24	**<0.001**	1.00
Social Support	0.04	6.73	**<0.001**	0.99
Treatment Adherence	0.02	2.89	**0.022**	0.78

*Controlling for sex, age, education level, time since diagnosis and severity of disease perception.

^a^The observed power gives the probability that the F test will detect the differences between groups equal to those implied by sample difference.

The results, presented in Table 4, showed that dispositional optimism, positive and negative affect, spirituality, and treatment adherence, were associated with general well-being (all p < 0.01); positive and negative affect, social support and treatment adherence were associated with physical health component (all p < 0.01); dispositional optimism, positive and negative affect, social support and treatment adherence were associated with mental health; and all psychosocial variables were associated with subjective well-being (all p < 0.01).

**Table 4:** Factors associated with HRQL and with SWB and parameter estimates of MANCOVA analysis

		**Parameter estimates**			
		**F**_**(1, 670)**_	**b (se)**	**t**	**p**
**QoL - General Well-being**	Dispositional Optimism	12.20	0.56 (0.16)	3.49	**0.001**
	Positive Affect	44.94	5.54 (0.79)	6.70	**<0.001**
	Negative Affect	78.87	−7.32 (0.82)	−8.88	**<0.001**
	Spirituality	8.44	2.28 (0.79)	2.91	**0.004**
	Social Support	1.28	0.03 (0.03)	1.13	0.259
	Treatment Adherence	6.20	2.56 (1.03)	2.49	**0.013**
**QoL - Physical Health**	Dispositional Optimism	1.79	−0.04 (0.03)	−1.34	0.181
	Positive Affect	7.51	−0.40 (0.15)	−2.74	**0.006**
	Negative Affect	15.89	0.60 (0.15)	3.99	**<0.001**
	Spirituality	2.10	0.21 (0.14)	1.45	0.148
	Social Support	9.32	−0.02 (0.01)	−3.05	**0.002**
	Treatment Adherence	8.39	−0,55 (0.19)	−2.89	**0.004**
**QoL - Mental Health**	Dispositional Optimism	5.29	−0.05 (0.02)	−2.29	**0.022**
	Positive Affect	20.40	−0.45 (0.10)	−4.52	**<0.001**
	Negative Affect	118.48	1.42 (0.10)	13.73	**<0.001**
	Spirituality	2.94	−0.17 (0.0)	−1.72	0.087
	Social Support	7.43	−0.01 (0.01)	−2.73	**0.007**
	Treatment Adherence	5.27	−0.22 (0.12)	−2.25	**0.022**
**Subjective Well-being**	Dispositional Optimism	4.94	0.35 (0.16)	2.22	**0.027**
	Positive Affect	17.41	3.29 (0.79)	4.17	**<0.001**
	Negative Affect	47.09	−5.59 (0.81)	−6.86	**<0.001**
	Spirituality	67.57	6.38 (0.78)	8.22	**<0.001**
	Social Support	17.88	0.12 (0.03)	4.23	**<0.001**
	Treatment Adherence	4.56	2.17 (1.05)	2.14	**0.033**

More positive affect and better treatment adherence contributed to better general well-being, physical and mental health and subjective well-being. Dispositional optimism and spirituality enhanced the general well-being and subjective well-being of the patients. Results also indicated that dispositional optimism had a statistically significant positive impact on mental health. Good social support contributed to better physical and mental health, as well as improved subjective well-being. Negative affect behaved as a statistically significant negative predictor for all components of HRQL and SWB.

### Subgroups of chronic diseases

Table 5 presents the descriptive statistics, mean (standard deviation), and correlations between psychosocial and the outcome variables (HRQL components and SWB), according to the subgroup of disease.

**Table 5:** Descriptive statistics and correlations between psychosocial variables and HRQL components and SWB, for subgroups of disease

	**Mean (SD)**			**GWB**			**MH**			**PH**			**SWB**		
	**M**	**N**	**C**	**M**	**N**	**C**	**M**	**N**	**C**	**M**	**N**	**C**	**M**	**N**	**C**
**Psychosocial Variables**															
Disp. Optimism (DOp)	20.1 (4.14)	20.77 (4.47)	21.61 (3.54)	0.40*	0.40*	0.35*	0.37*	0.41*	0.35*	0.18*	0.22*	0.23*	0.40*	0.40*	0.44*
Positive Affect (PA)	2.86 (0.79)	3.01 (0.79)	3.02 (0.76)	0.44*	0.27*	0.43*	0.33*	0.33*	0.32*	0.25*	0.097	0.33*	0.35*	0.27*	0.41*
Negative Affect (NA)	2.08 (0.82)	1.83 (0.68)	1.86 (0.66)	−0.47*	−0.51*	−0.44*	−0.59*	−0.61*	−0.50*	−0.34*	−0.40*	−0.34*	−0.42*	−0.35*	−0.41*
Spirituality (Sp)	2.65 (0.77)	2.63 (0.74)	2.88 (0.71)	0.21*	0.13	0.16*	0.16*	0.06	0.20^&^	0.01	−0.05	0.09	0.42*	0.30*	0.48*
Social Support (SS)	66.74 (21.44)	71.34 (20.37)	69.10 (21.97)	0.37*	0.12	0.30*	0.35*	0.21*	0.31*	0.28*	−0.004	0.29*	0.48*	0.25*	0.27*
Treatment Adherence (TA)	5.22 (0.59)	5.51 (0.44)	5.64 (0.41)	0.15*	0.06	0.22*	0.18*	0.22*	0.16*	0.18*	0.16*	0.17*	0.14*	0.12	0.60&
**Quality of Life Variables**															
General well-being (GWB)	45.59 (19.21)	52.01 (16.78)	55.17 (18.60)	-	-	-	0.68*	0.58*	0.66*	0.71*	0.64*	0.74*	0.56*	0.50*	0.54*
Mental health (MH)	62.63 (25.90)	70.46 (22.82)	69.98 (24.97)	0.71*	0.58*	0.66*	-	-	-	0.64*	0.60*	0.67*	0.51*	0.44*	0.48*
Physical health (PH)	59.83 (28.14)	68.06 (24.26)	67.37 (24.41)	0.68*	0.64*	0.74*	0.64*	0.60*	0.67*	-	-	-	0.39*	0.41*	0.49*
Subjective well-being (SWB)	60.42 (17.84)	65.27 (15.50)	67.52 (16.16)	0.56*	0.50*	0.54*	0.51*	0.44*	0.48*	0.39*	0.41*	0.49*	-	-	-

*p<0.001.

^&^p<0.05.

M – Metabolic Subgroup; N – Neurologic Subgroup; C – Cancer Subgroup.

Generally, all psychosocial variables have a statistically significant association with outcome variables, in all subgroups of disease. For each subgroup of disease patients (metabolic, neurologic and cancer) negative affect showed a statistically significant inverse relation with HRQL components and SWB. For all subgroups of disease, only spirituality did not show a statistically significant association with physical health.

For each subgroup of diseases (Table 6), most of the psychosocial variables were significantly associated with quality of life and subjective well-being, controlling for socio-demographic and clinical variables.

**Table 6:** Factors associated with HRQL and with SWB: results of multivariate tests of MANCOVA analysis for subgroups of disease*

	**Value Roy’s largest root**			**F**_**(4, 667)**_	**F**_**(4, 307)**_	**F**_**(4, 667)**_	**p**			**Observed power**		
	**M**	**N**	**C**	**M**	**N**	**C**	**M**	**N**	**C**	**M**	**N**	**C**
Dispositional Optimism	0.02	0.04	0.19	1.74	1.76	0.77	0.142	0.14	0.55	0.53	0.53	0.24
Positive Affect	0.09	0.09	0.12	7.23	4.00	4.36	**<0.001**	**0.004**	**0.002**	0.99	0.90	0.93
Negative Affect	0.36	0.03	0.26	27.97	16.79	10.53	**<0.001**	**<0.001**	**<0.001**	1.00	1.00	1.00
Spirituality	0.09	0.09	0.18	6.76	3.92	7.49	**<0.001**	**0.005**	**<0.001**	0.99	0.89	0.99
Social Support	0.10	0.05	0.09	7.73	1.99	3.85	**<0.001**	0.098	**0.005**	0.99	0.59	0.89
Treatment Adherence	0.03	0.03	0.01	2.53	1.29	0.41	**0.041**	0.274	0.804	0.71	0.40	0.14

*Controlling for sex, age, education level, time since diagnostics and severity of disease perception.

M – Metabolic Subgroup; N – Neurologic Subgroup; C – Cancer Subgroup.

#### Chronic metabolic disease

Results presented in Table 7 for the metabolic disease subgroup showed that an optimistic attitude contributes to better general well-being (p = 0.021). Positive and negative affect had a positive and negative, respectively, statistically significant association with all components of HRQL and SWB (all p < 0.01). More social support also contributed to better physical (p = 0.023) and mental health (p = 0.025) and improved subjective well-being (p < 0.001). Adequate treatment adherence for patients with metabolic diseases contributed to better physical health (p = 0.002).

**Table 7:** Factors associated with HRQL and with SWB and parameters estimates of MANCOVA analysis for subgroups of disease

**Parameter estimates**													
		**F(1, 370)**	**F(1, 170)**	**F(1, 166)**	**b (se)**			**t**			**p**		
		**M**	**N**	**C**	**M**	**N**	**C**	**M**	**N**	**C**	**M**	**N**	**C**
**QoL - General Well-being**	Dispositional Optimism	5.44	6.54	2.48	0.56 (0.24)	0.68 (0.27)	0.56 (0.35)	2.31	2.56	1.58	**0.021**	**0.011**	0.117
	Positive Affect	26.19	9.83	7.53	6.14 (1.20)	4.19 (1.34)	4.51 (1.64)	5.12	3.14	2.74	**<0.001**	**0.02**	**0.007**
	Negative Affect	28.67	40.02	13.52	−6.09 (1.14)	−9.71 (1.54)	−6.96 (1.89)	−5.36	−6.33	−3.86	**<0.001**	**<0.001**	**<0.001**
	Spirituality	3.79	0.03	4.13	2.29 (1.18)	0.25 (1.43)	3.29 (1.62)	1.95	0.18	2.03	0.052	0.862	**0.044**
	Social Support	2.82	0.02	0.18	0.07 (0.04)	−0.01 (0.05)	0.02 (0.05)	1.68	−0.15	0.42	0.094	0.879	0.673
	Treatment Adherence	2.28	1.56	1.36	2.12 (1.40)	2.76 (2.21)	3.08 (2.64)	1.51	1.25	1.17	0.132	0.213	0.244
**QoL - Physical Health**	Dispositional Optimism	0.39	1.52	0.75	−0.02 (0.04)	−0.06 (0.05)	−0.07 (0.09)	−0.63	1.23	−0.87	0.529	0.219	0.387
	Positive Affect	4.47	0.92	2.60	−0.38 (0.18)	−0.02 (0.23)	−0.64 (0.39)	−2.11	−0.96	−1.61	**0.035**	0.339	**0.015**
	Negative Affect	12.28	20.17	<0.001	0.60 (0.17)	1.19 (0.26)	<0.001 (0.46)	3.50	4.49	<0.001	**0.001**	**<0.001**	1.000
	Spirituality	0.22	1.23	1.09	0.08 (0.17)	0.27 (0.25)	0.40 (0.39)	0.46	1.11	1.04	0.643	0.269	0.298
	Social Support	5.25	1.83	11.86	−0.01 (0.01)	0.11 (0.01)	−0.04 (0.01)	−2.29	1.35	−3.44	**0.023**	0.178	**0.001**
	Treatment Adherence	9.91	4.57	0.81	−0.67 (0.21)	−0.82 (0.38)	−0.57 (0.64)	−3.15	−2.14	−0.89	**0.002**	**0.034**	0.371
**QoL - Mental Health**	Dispositional Optimism	1.21	2.40	1.85	−0.03 (0.03)	−0.06 (0.04)	−0.07 (0.05)	−1.10	−1.55	−1.34	0.272	0.123	0.176
	Positive Affect	10.31	7.90	3.33	−0.46 (0.14)	−0.05 (0.19)	−0.04 (0.22)	−3.21	−2.81	−1.82	**0.001**	**0.006**	**0.007**
	Negative Affect	99.35	52.97	37.85	1.36 (0.14)	1.55 (0.21)	1.55 (0.25)	9.97	7.28	6.15	**<0.001**	**<0.001**	**<0.001**
	Spirituality	0.36	0.01	3.96	−0.09 (0.14)	−0.02 (0.19)	−0.43 (0.22)	−0.60	−0.11	−1.99	0.549	0.911	**0.048**
	Social Support	5.10	1.72	2.22	−0.01 (0.01)	0.01 (0.01)	−0.01 (0.01)	−2.26	−1.31	−1.49	**0.025**	0.192	0.138
	Treatment Adherence	2.09	3.18	0.50	−0.24 (0.17)	−0.55 (0.31)	−0.25 (0.35)	−1.45	−1.78	−0.71	0.149	0.076	0.481
**Subjective Well-being**	Dispositional Optimism	2.38	1.29	0.95	0.37 (0.24)	0.34 (0.29)	0.32 (0.32)	1.54	1.13	0.98	0.121	0.258	0.330
	Positive Affect	4.01	3.17	15.75	2.39 (1.19)	2.67 (1.50)	5.89 (1.48)	2.00	1.78	3.97	**0.046**	0.077	**<0.001**
	Negative Affect	24.09	12.03	8.87	−5.54 (1.13)	−5.97 (1.72)	−5.08 (1.71)	−4.91	−3.47	−2.98	**<0.001**	**0.001**	**0.003**
	Spirituality	21.08	9.40	25.98	5.37 (1.17)	4.92 (1.60)	7.46 (1.46)	4.59	3.07	5.09	**<0.001**	**0.003**	**<0.001**
	Social Support	28.84	0.25	0.16	0.23 (0.04)	0.03 (0.05)	0.02 (0.05)	5.37	0.49	0.40	**<0.001**	0.621	0.688
	Treatment Adherence	0.56	1.18	0.52	1.04 (1.39)	2.69 (2.49)	1.71 (2.38)	0.75	1.09	0.72	0.456	0.279	0.473

M – Metabolic Subgroup; N – Neurologic Subgroup; C – Cancer Subgroup.

#### Chronic neurologic disease

In patients with chronic neurologic diseases, negative affect had a statistically significant negative impact in all components of HRQL and SWB (all p < 0.01). Positive affect was statistically associated with better general well-being (p = 0.02) and improved mental health (p = 0.006). More optimistic attitude developed better general well-being (p = 0.011), adequate treatment adherence contributed to enhanced physical health (p = 0.034), and feel-good spirituality enhanced subjective well-being (p = 0.003) (Table 7).

#### Cancer chronic disease

For the cancer chronic disease subgroup, positive affect and spirituality showed a statistically significant association with general well-being and mental health components, and with subjective well-being (all p < 0.01). More optimistic patients and those with feel-good spirituality had better quality of life (in these domains). Negative affect had a negative, statistically significant, impact, and contributed to a negative predictor for general well-being (p < 0.001), mental health (p < 0.001) and subjective well-being (p = 0.003). Positive affect (p = 0.015) and more social support (p = 0.001) contributed to improved physical health (Table 7).

## Discussion

The purpose of the present study was to explore the role of psychosocial factors in predicting quality of life in chronic Portuguese patients, controlling for socio-demographic and clinical variables. This study included a set of chronic disease patients and involved a variety of psychosocial variables. Several previous studies included only one specific disease and one psychosocial variable [11,20,21,42].

To summarize our findings, dispositional optimism, positive affect, spirituality, social support and treatment adherence are significant positive psychosocial predictors of quality of life.

Positive and negative affect have a significantly positive and negative correlation with HRQL and with SWB, respectively. Similar findings are reported by Singh and Jha [13], especially regarding SWB. This study supports similar investigations, which also observed that positive affect was the strongest predictor of global well-being, and that life satisfaction was a function of the preponderance of positive affect in daily life. Dispositional optimism also had a positive correlation with HRQL and with SWB. Spirituality only had a positive significant moderate correlation with SWB. The results from our study are consistent with those reported by investigators studying cancer patients [43].

Furthermore, the findings of this study suggest that dispositional optimism associated with general and SWB and also exerts a statistically significant positive effect in mental health. Optimism may significantly influence mental and physical well-being [44] by the promotion of a healthy lifestyle, as well as adaptive behaviours and cognitive responses associated with greater flexibility, problem-solving capacity, and a more efficient treatment of negative information [44]. These results are consistent with previous studies [19,20] in which higher levels of optimism were prospectively associated with increased SWB.

More positive affect and adequate treatment adherence are associated with a better HRQL and a better SWB. Negative affect behaves as a negative predictor of these components. In previous research an association between positive and negative affect and quality of life has been found: in a group of chronic patients (with arthritis, cardiovascular disease, chronic obstructive pulmonary disease or diabetes), high positive affect and low negative affect were associated with higher physical and mental health [45]. Similar results were found with blue-collar women, with positive affect being related to women's self-reported health and exercise. Alternatively, negative affect was strongly correlated with complaints in a wide range of health problems [21].

Results also showed that spirituality was associated to a better HRQL and SWB. Visser, Garssen and Vingerhoets [43] stated that spirituality was defined as an experience of a connection with the essence of life. These authors found a positive association between spirituality and well-being in a majority of cancer cross-sectional studies. In a rheumatoid arthritis study, authors verified that spirituality may facilitate and improve emotional status and resilience, by experiencing more positive feelings and attending to the positive elements of life [46].

Another finding was that good social support was associated with better physical and mental health and to better SWB. Yang and al. [47] also demonstrated that social support has a positive relationship with physical and psychological well-being. Controlling for demographic variables, Tang et al. [42] found that in a group of diabetes patients social support was a positive predictor of quality of life. The positive relationship between social support and HRQL, in cancer patients underscores the importance of social support as a beneficial resource in sustaining an acceptable HRQL [48].

This study analysed simultaneously and found associations between psychosocial variables and HRQL, in different subgroups of chronic diseases treated together (metabolic, neurologic and cancer). We found that the three subgroups had similar psychosocial predictors of HRQL. In all subgroups, more positive affect was associated with better general well-being and mental health. People that feel-good spiritually experience had better SWB. In the metabolic and neurologic chronic disease subgroup, it was found that adequate treatment adherence were associated with better physical health. Social support was a better predictor of physical health in the metabolic and cancer disease subgroup. In general, in all subgroups, negative affect behaves as a negative predictor of HRQL.

The predictors of HRQL for all groups of chronic diseases are similar to those found in different chronic disease subgroups: positive affect, social support, treatment adherence and spirituality. Patients who had more positive affect, additional social support, and adequate treatment adherence or feel-good spirituality, handled disease conditions better and consequently had a better HRQL.

## Conclusions

This work was an attempt to identify psychosocial factors associated with HRQL in persons with chronic diseases. This study contributed to understanding and improving the processes associated with HRQL, which is relevant for health care providers, and chronic diseases support. A better understanding of the psychosocial factors that simplify the daily lives of patients ought to lead to better control of the disease, which should lead to better outcomes for patients, and reduced treatment costs.

## Abbreviations

GWB: General well-being; IQOLA: International quality of life assessment; LOT-R: Life orientation test-revised; MANCOVA: Multivariate analysis of covariance; MOS: Medical outcomes study; MH: Mental health; NA: Negative affect; PA: Positive affect; PANAS: Positive and negative affect schedule; PH: Physical health; HRQL: Quality of life; SF-36: Short-form health survey; SWB: Subjective well-being.

## Competing interests

The authors declare that they have no competing interests.

## Authors’ contributions

EV was involved in the design of the study, performed the statistical analyses and drafted the manuscript. DM was involved in the discussion of statistical analysis contents and in the revision of the manuscript for intellectual contents. JPR conceived the study and was involved in the revision of the manuscript for intellectual contents. IS, LP, RM, HC and AMS were involved the recruitment of patients and collection of clinical data. All authors approved the final manuscript.
